# Effects of rotigotine on sleep in Parkinson’s disease patients: a Parkinson’s KinetiGraph study

**DOI:** 10.3389/fneur.2025.1591537

**Published:** 2025-05-27

**Authors:** Sotirios Grigoriou, Carin Janz, Malcolm Horne, Filip Bergquist, Nil Dizdar, Per Odin

**Affiliations:** ^1^Division of Neurology, Department of Clinical Sciences Lund, Lund University, Lund, Sweden; ^2^Department of Neurology, Rehabilitation Medicine, Memory and Geriatrics, Skane University Hospital, Lund, Sweden; ^3^Department of Medicine, St Vincent’s Hospital, University of Melbourne, Melbourne, VIC, Australia; ^4^Division of Neurology, Sahlgrenska University Hospital, Gothenburg, Sweden; ^5^Department of Pharmacology, University of Gothenburg, Gothenburg, Sweden; ^6^Department of Biomedical and Clinical Sciences, Linkoping University, Linköping, Sweden

**Keywords:** Parkinson’s disease, Parkinson’s KinetiGraph, rotigotine, sleep disturbances, Parkinson disease sleep scale-2, sleep quality, daytime sleepiness

## Abstract

**Introduction:**

Recent studies suggest that the dopamine agonist (DA) rotigotine improves sleep among Parkinson disease (PD) patients. Parkinson’s KinetiGraph (PKG) offers a home-based alternative for evaluating sleep. We investigated the effect of rotigotine on sleep in PD patients with PKG and questionnaires. Secondarily, the effects of rotigotine on daytime sleepiness, motor symptoms, quality of life and correlations between PKG variables and rating scale results were investigated.

**Method:**

Thirty-two PD patients with sleep disturbances (Clinical Global Impression-Severity (CGI-S) ≥ 3) were included in this observational study. Before start of treatment and during stable dose with rotigotine patients were assessed with Parkinson’s disease sleep scale 2 (PDSS-2), Epworth Sleepiness Scale (ESS), Parkinson’s disease quality of life questionnaire (PDQ-8), European Quality of life five dimensions (EQ-5D-5L) questionnaires and PKG recordings (24 h/day for 6 days). Clinicians evaluated sleep using CGI scales, and PD severity using Clinical Impression of Severity Index for Parkinson’s Disease (CISI-PD).

**Results:**

Rotigotine did not significantly improve total PDSS-2 or PKG nighttime scores in the entire group, but PDSS-2 improved among patients with PDSS-2 ≥ 18 at baseline and for DA-naïve patients (*p* = 0.009 and *p* = 0.013). Treatment improved percent time tremor (PTT; *p* < 0.001), percent time immobile during daytime (PTI_D_; *p* < 0.001), CISI-PD (*p* < 0.001), PDQ-8 (*p* = 0.014), and EQ-5D-5L (*p* = 0.002). No significant correlations were found between PTI_D_ and ESS-total (ρ = −0.046, *p* = 0.718) or between combined sleep score (CSS) and PDSS-2 total (ρ = −0.065, *p* = 0.612).

**Conclusion:**

Rotigotine improved sleep in patients with a baseline PDSS-2 ≥ 18 and in DA-naïve patients, but not in the whole study group. Additionally, rotigotine seemed to improve motor function and quality of life. PTI_D_ improved with treatment. Whether the improved PTI_D_ reflects a positive impact on daytime sleepiness or just improved mobility and to what extent PKG nighttime scores accurately represent sleep variables remains to be investigated in further studies.

## Introduction

Sleep disturbances and daytime sleepiness are common in patients with Parkinson disease (PD), can be disease- or treatment-related and significantly impact health related quality of life (1). Rotigotine, a non-ergot dopamine agonist (DA) delivered through a transdermal patch, provides stable plasma concentrations and consequently reduces motor fluctuations (2). It also shows promise in improving sleep-related issues, including night-time pain, nocturnal motor impairment (3), sleep fragmentation (4), and overall sleep quality (5). Furthermore, the Evidence Based Medicine in Movement Disorders committee considers rotigotine as possibly useful for improving sleep (6). In the RECOVER study, a four-week randomized controlled trial (RCT), rotigotine treatment led to improvements in 10 out of 15 items on the Parkinson’s disease sleep scale 2 (PDSS-2) (3). Long-term use of rotigotine demonstrated sustained effects on sleep and motor function in a one-year follow-up study (7). A recent meta-analysis has also concluded that rotigotine improves both motor symptoms and sleep quality among individuals with PD (8). Moreover, Calandra-Buonara et al. (5) found that rotigotine treatment had positive effects on nocturnal and diurnal sleep disturbances in PD patients, as per actigraphy recordings, although not reflected in ESS scores. However, in contrast to these studies, an RCT evaluating rotigotine’s effectiveness using the non-motor symptoms scale found no significant improvements in sleep or fatigue, as evaluated by the Non-Motor Symptoms Scale for Parkinson’s Disease (9). While antiparkinsonian treatment, especially DAs, can worsen excessive daytime sleepiness (EDS) (10), some studies have shown that rotigotine does not exacerbate daytime sleepiness (11–13), while others demonstrated an improvement in daytime sleepiness with rotigotine, as indicated by actigraphy and the Epworth sleepiness scale (ESS) (5, 14).

Polysomnography (PSG) is regarded as the gold standard for objective sleep measurement (15), but can be resource demanding and usually requires overnight hospital admission, which can impact sleep quality and make long-term monitoring challenging (16). The Parkinson’s KinetiGraph (PKG) is a measurement system used for ambulatory, objective assessment of PD motor symptoms, such as tremor, bradykinesia, dyskinesia, and motor fluctuations (17). Furthermore, studies have investigated the capability of PKG in assessing night sleep and daytime sleepiness in PD patients. McGregor et al. (18) found that PKG scores can differentiate with good sensitivity and specificity between normal and abnormal PSG studies, and that PKG provides a fairly accurate picture of wakefulness, sleep duration, sleep quality, and sleep fragmentation. Another study supports the use of PKG immobility and mobility segments as indicators of wakefulness and sleep and suggests that PKG can be used as a rough evaluation of night-time sleep and as a tool to determine whether PSG is needed (19). Kotschet et al. (20) found a correlation between daytime percent time immobile (PTID) measured with PKG and ESS, suggesting that it is a useful measure of daytime sleep, or at least somnolence in PD patients. However, Höglund et al. (21) could not detect any significant correlations between PTI and diary reported daytime sleepiness.

Most studies investigating the effects of rotigotine on sleep have relied on questionnaires (3, 4, 7, 8, 14), with only a few utilizing more objective methods (5, 22, 23). Therefore, further research is needed to objectively confirm rotigotine’s suggested sleep-enhancing effects.

The primary objective of this study was to investigate the effects of rotigotine on sleep in PD patients using well established rating scales and PKG recordings. Secondarily, it aimed to investigate the impact of rotigotine treatment on daytime sleepiness, quality of life and motor symptoms in PD patients. Furthermore, the study aimed to explore the correlations between PKG parameters used to evaluate sleep and daytime sleepiness, and corresponding questionnaires that evaluate these same parameters.

## Materials and methods

This investigator-initiated, observational, prospective study is a collaboration within the Swedish Parkinson Research Network. Forty patients were included in the study and 32 patients completed the study.

### Participation criteria

PD patients aged 18–85 who were experiencing sleep disturbances (Clinical Global Impression-Severity (CGI-S) ≥ 3) were included. The CGI-S scale was used as a quick and broad screening tool to capture both mild and severe sleep disturbances, allowing for inclusion of patients that more specific scales might miss. Its use also aligns with previous studies on rotigotine and sleep, ensuring comparability (3, 4). Participants were required to have maintained a stable PD treatment regimen for at least 28 days. Patients were excluded according to the following criteria: advanced therapies such as deep brain stimulation, apomorphine infusion, or levodopa infusion; oral DA administration the last 28 days; dementia or significant cognitive impairment; clinically significant prostate problems causing sleep problems, sleep apnea syndrome, or other diagnosed non-PD-related conditions significantly impacting nocturnal sleep. Initiation or modification of sedatives or hypnotics during the study was not permitted.

### Study design

**Figure 1:**
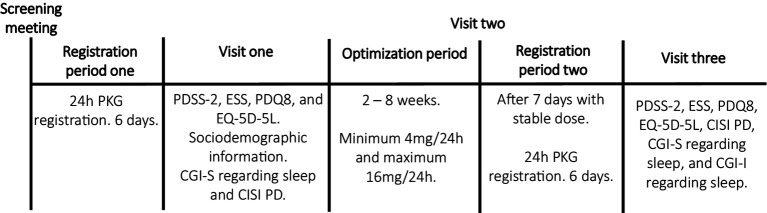
Explained study design. PKG, Parkinson’s KinetiGraph; PDSS-2, Parkinson’s disease sleep scale 2; ESS, Epworth Sleepiness Scale; PDQ8, Parkinson’s disease quality of life questionnaire; EQ-5D-5L, European Quality of life five dimensions with five levels; CGI-S, Clinical Global impressions severity; CISI-PD, Clinical Impression of Severity Index for Parkinson’s Disease; CGI-I, Clinical Global impressions improvement.

The study flow chart is presented in Figure 1.

PD patients with sleep disturbances, already scheduled to start rotigotine treatment, were evaluated based on eligibility criteria. Demographic and clinical data were gathered, and levodopa equivalent doses (LED) were computed following Tomlinson et al. (24). Before initiating rotigotine, eligible patients underwent the first PKG recording, wearing the device continuously on their most affected side for 24 h over six consecutive days. After this recording, participants completed the PDSS-2 (25), ESS (26), Parkinson’s disease quality of life questionnaire (PDQ-8) (27), and European Quality of life five dimensions (EQ-5D-5L) questionnaires (28). Study clinicians also assessed the patients’ sleep through interviews using the CGI-S and evaluated disease severity using the Clinical Impression of Severity Index for Parkinson’s Disease (CISI-PD) (29).

Study clinicians titrated and optimized rotigotine per the study protocol’s titration plan, starting patients at 2 mg with weekly increases of 2 mg. Weekly follow-ups in-person or by phone, aimed to identify a dose that provided adequate motor symptom relief without bothersome side effects. If side effects occurred, the dose was reduced by 2 mg to the last tolerable level. The up-titration rate could be slightly adjusted based on factors like tolerance, prior medications, and clinical response, with target dosages ranging from 4 mg to 16 mg (2, 30). After maintaining the achieved dose for at least 1 week, another PKG recording period followed. Following this second recording, the clinician evaluated the patient’s sleep improvement using CGI - Improvement (CGI-I) scale (29), and all questionnaires and clinical scales that were assessed at baseline were completed again.

### The PKG system and glossary of PKG terms

The PKG, developed by Global Kinetics Corporation, is a wrist-worn device worn for up to 10 days (18), using accelerometer data to generate continuous variables analyzed by specialized algorithms (31). For this study, patients wore the PKG on their most affected side for 6 days. A detailed glossary including all presented PKG variables are available in the Supplementary material.

#### Percent time immobile

PTI_D_ is a measure of daytime immobility that has shown concordance with the detection of daytime sleep by PSG (20). High PTI values have been associated with higher ESS scores in PD patients (20).

#### Combined sleep score

Combined sleep score (CSS) quantifies sleep quality by normalizing and scoring nighttime PTI, PTS_N_ (percent time sleep during night-time), and SQ (sleep quality) variables. Higher values indicate better sleep, and CSS has been shown to a broad inverse correlation with PDSS-2 (18).

### Statistical analyses

Co-author MH compiled the PKG data. Descriptive statistics are presented with median and interquartile ranges (IQR) or mean and range. Since the data was not normally distributed, changes in PKG variables and questionnaires before and with treatment were assessed using the Wilcoxon signed rank test. Two-sided *p*-values were used, and statistical significance was defined as *p* ≤ 0.05. Since the data was not normally distributed, the Spearman correlation test was used to investigate the correlation between PTI_D_ and ESS, as well as between PDSS-2 and CSS. Statistical analyses were performed using SPSS Statistics version 29 (IBM Corp) and Microsoft Excel version 365 (Microsoft Corporation). Graphs were created using GraphPad Prism version 9.0 (GraphPad Software, Inc).

#### Primary outcomes

The primary outcomes of the study were changes in total PDSS-2 and CSS; *p*-value was adjusted to 0.025 after Bonferroni correction.

#### Secondary outcomes

Secondary outcomes and post-hoc analyses were considered exploratory and therefore we did not correct for multiple comparisons (32). Secondary outcomes were the CGI-I regarding sleep, the remaining daytime and nighttime PKG scores, the ESS, the CISI-PD questionnaire, the PDQ-8, the EQ-5D-5L questionnaire, and analyses of correlations between PDSS-2 and CSS as well as between ESS and PTI_d._ Post-hoc analyses examined the effects of baseline PDSS-2 and previous history of DA use on PDSS-2 outcomes.

## Results

### Participant characteristics

**Figure 2:**
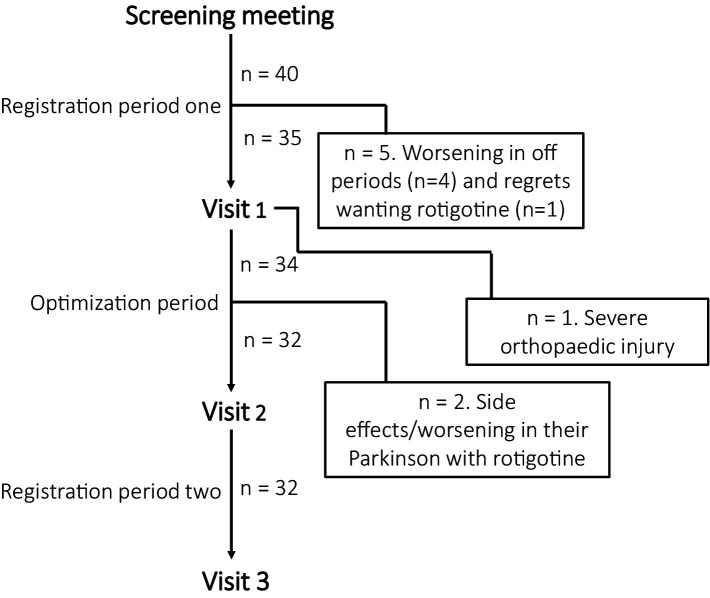
Participant inclusion and discontinuation reasons. During registration period one, four participants discontinued due to worsening off periods without dopamine agonist and before starting rotigotine. One patient was excluded during visit 1 due to a severe orthopedic injury that affected their sleep. During optimization period, one participant discontinued the study due to skin rash, and another experienced worsened PD symptoms with rotigotine.

Figure 2 illustrates participant inclusion and discontinuation. Of 40 participants, 32 completed the study: 29 from Skane University Hospital, one from Sahlgrenska University Hospital, and two from Linköping University Hospital. All patients were on concomitant anti-parkinsonian medication. Sixteen patients were DA-naïve, while 16 had prior DA treatment, which was discontinued for at least 28 days prior to study inclusion. Baseline evaluations were conducted without DA treatment. The mean age was 67 years (range: 50–82), mean LED was 783 mg (range: 120–1983), mean time since diagnosis was 5 years (range: 0–14), and the median baseline CGI-S score for sleep was 4 (IQR: 4–5).

### Rotigotine usage and side effects

The mean maintenance rotigotine dose was 5 mg (range: 4–8) (Table 1). The average time on maintenance dose before finishing the second PKG registration was 16 days (range: 13–32), with a total treatment duration averaging 29 days (range: 16–49). Reported rotigotine side effects included nausea (*n* = 4), headache (*n* = 1), worsened tremor (*n* = 1), skin irritation/rash (*n* = 3), and worsened dyskinesia (*n* = 1).

**Table 1:** Demographic and clinical characteristics^a^.

Male/female (*n*, %)	21 (66%)/11 (34%)
Age, years (mean, range)	67 (50–82)
Age at PD onset, years (mean, range)	60 (44–76)
Age at PD diagnosis, years (mean, range)	62 (46–77)
Duration of PD, years since diagnosis (mean, range)	5 (0–14)
Rotigotine maintenance dose, mg/24 h (mean, range)	5 (4–8)
Days with rotigotine maintenance dose^b^ (mean, range)	16 (13–32)
Total days with rotigotine^c^ (mean, range)	29 (16–49)
LED^d^ (mean, range)	783 (120–1,983)
Hoehn and Yahr stage (median, IQR)	2 (2–2)
Stage 1 (*n*, %)	6 (19%)
Stage 2 (*n*, %)	15 (47%)
Stage 3 (*n*, %)	6 (19%)
Not registered (*n*, %)	5 (16%)
CGI-S regarding sleep (screening)^e^	4 (4–5)
CGI-S 3 (*n*, %)	3 (9%)
CGI-S 4 (*n*, %)	17 (53%)
CGI-S 5 (*n*, %)	8 (25%)
CGI -S 6 (*n*, %)	4 (13%)
PDSS-2^f^ total score at baseline (median, IQR)	17 (13–24)

a Presented as mean (range), median (IQR, interquartile range) or number and percentages.

b Time with rotigotine maintenance dose (optimal dose) until finishing the second PKG registration period.

c Time from starting rotigotine treatment until finishing the second PKG registration period.

d LED, Levodopa equivalent dose, mg/day.

e Clinical Global Impression-Severity regarding sleep. 3 = mildly ill, 4 = moderately ill, 5 = markedly ill, 6 = severely ill.

f Parkinson disease sleep scale 2.

### Sleep and daytime sleepiness

#### Sleep

**Figure 3:**
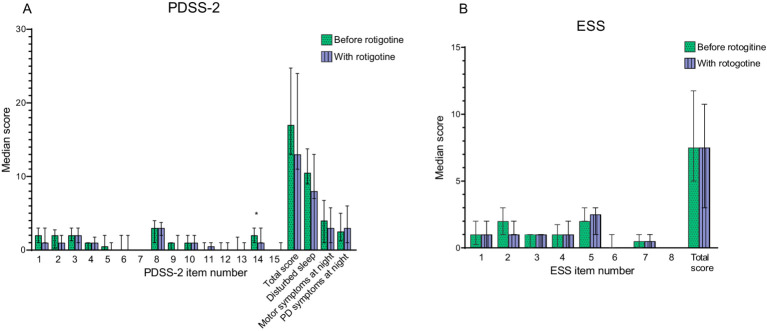
Sleep and daytime sleepiness before and with rotigotine treatment according to questionnaires. Median values are presented, and error bars represent the interquartile range. Green bars with dots indicate scores before rotigotine treatment, while purple bars with lines indicate scores with rotigotine treatment. The total score is the sum of all item scores. Statistical significance is denoted as *p ≤ 0.05, calculated using the Wilcoxon signed rank test. Bonferroni adjusted *p* value was set as *p* ≤ 0.025 for PDSS-2 **(A)** Parkinson’ s disease sleep scale (PDSS-2). Disturbed sleep include items 1, 2, 3, 8, and 14. Motor symptoms at night include items 4, 5, 6, 12, and 13. PD symptoms at night includes items 7, 9, 10, 11, and 15. Significant improvement was observed in question 14 (sleepiness after waking, *p* = 0.02) **(B)** Epworth Sleepiness Scale (ESS).

There was no significant improvement in PDSS-2 total score, with a median of 17 (IQR: 13–24) without rotigotine treatment and 13 (IQR: 11–24) with rotigotine treatment (*p* = 0.13) (Figure 3). Significant improvement was observed in PDSS-2 item 14 (sleepiness after waking) with a median of 2 (IQR: 1–3) before treatment and 1 (IQR: 1–3) with treatment (*p* = 0.02).

Among the 15 patients with clinically significant sleep disturbances (PDSS-2 ≥ 18) before rotigotine treatment (33), the PDSS-2 total score improved from a median of 25 (IQR: 21–27.5) to a median of 20 (IQR: 12–25) (*p* = 0.009). PDSS-2 subtotals for disturbed sleep (items 1, 2, 3, 8 and 14) improved from a median of 13 (IQR: 11.5–14.5) to a median of 8 (IQR: 8–13) (*p* = 0.013), and subtotals for PD symptoms at night (items 7, 9, 10, 11, and 15) improved from a median of 5 (IQR: 4–8.5) to a median of 4 (IQR: 2.5–7) (*p* = 0.041). All participants were evaluated without DA treatment at baseline, though 16 of them had previously been treated with DA. Explorative subgroup analysis showed that DA- naïve patients improved significantly in total PDSS-2 from a median of 17.5 (IQR: 13–25) to 12.5 (IQR:10–23) (*p* = 0.013) with rotigotine treatment, while no significant improvement was detected for patients previously on oral DA.

**Figure 4:**
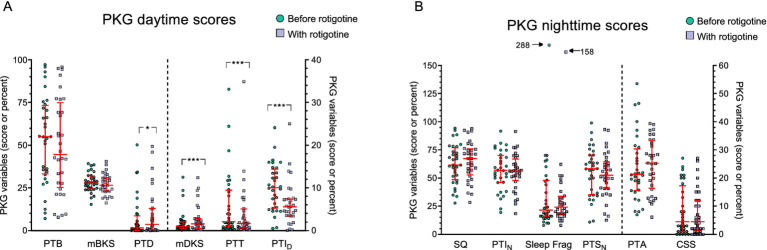
PKG scores before and with Rotigotine treatment. Median values are presented, and error bars represent the interquartile range. Green circles indicate scores before rotigotine treatment, while purple squares indicate scores with rotigotine treatment. The left side of the figures is separated by a dotted line and corresponds to the left Y axis, while the right side relates to the right Y axis. Statistical significance is denoted as *p ≤ 0.05, ***p ≤ 0.001, calculated using the Wilcoxon signed rank test. **(A)** Daytime PKG scores, assessed from 09:00 to 18:00, include the following parameters: PTB, percent time in bradykinesia; mBKS, median bradykinesia score during daytime; PTD, percent time in dyskinesia; mDKS, median dyskinesia score; PTT, percent time in tremor; PTI_D_, percent time immobile during daytime. PTT and PTI_D_ showed a significant improvement with rotigotine (*p* < 0.001 and *p* < 0.001), while PTD and mDKS demonstrated a significant deterioration with treatment (*p* = 0.016 and *p* = 0.001). **(B)** Night-time PKG scores, assessed from 23:00 to 06:00, include the following parameters: SQ, sleep quality; PTI_N_, percent time immobile during night-time; Sleep Frag, Sleep fragments (the median duration of sleep fragments in minutes); PTS_N_, percent time asleep during night-time; PTA, Night percent time active score; CSS, Combined sleep score.

The median score on CGI-S regarding sleep improved from 4 to 3 (*p* < 0.001) with rotigotine treatment. According to CGI-I for sleep; one patient improved very much, five improved much, 13 improved minimally, eight did not change, two got minimally worse and three got much worse. No significant improvement was observed in the PKG nighttime scores (Figure 4B).

#### Daytime sleepiness

Median ESS total score was 7.5 both before and with rotigotine treatment (IQR before: 5–11.3; IQR after: 3–10.3) (*p* = 0.358) (Figure 3). Figure 4A presents the daytime PKG scores. Based on PKG data, significant improvements were observed in PTI_D_ with a median of 10 (IQR: 5–14) before rotigotine and a median of 6 (IQR: 3–7) with rotigotine (*p* < 0.001). Twelve patients transitioned from high to normal PTI_D_ scores (target score ≤10%) (34).

### Motor symptoms and quality of life

#### Quality of life

**Figure 5:**
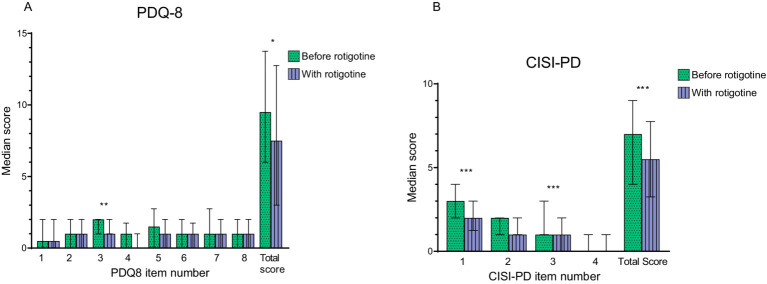
Motor complications and quality of life before and with rotigotine treatment according to questionnaires. Median values are presented resented, and error bars represent the interquartile range. Green bars with dots indicate scores before rotigotine treatment, while purple bars with lines indicate scores with rotigotine treatment. The total score is the sum of all item scores. Statistical significance is denoted as *p ≤ 0.05, **p ≤ 0.01, ***p ≤ 0.001, calculated using the Wilcoxon signed rank test. **(A)** Parkinson’s Disease Quality of Life Questionnaire (PDQ-8). Significant improvements were observed in question 3 (felt depressed, *p* = 0.07), and the total score (*p* = 0.014). **(B)** Clinical Impression of Severity Index for Parkinson’s Disease (CISI-PD). Significant improvements were observed in question 1 (motor signs), question 3 (motor complications including dyskinesia and fluctuations), and the total score (*p* = 0.001, *p* < 0.001, and *p* < 0.001).

Median PDQ-8 score improved from 9.5 (IQR: 6–13) to 7.5 (IQR: 3–12) (*p* = 0.014), particularly in terms of decreased feelings of depression (question 3 on PDQ-8, *p* = 0.007) (Figure 5). The median EQ-5D-5L Time Trade Off (TTO) improved from 0.79 (IQR: 0.7–0.9) to 0.84 (IQR: 0.8–0.9) (*p* = 0.002), and the median VAS score improved from a median of 60 (IQR: 44.5–70) to 67.5 (IQR: 52–76) (*p* = 0.002).

#### PD severity

Median CISI-PD score improved from 7 (IQR: 4–9) to 5.5 (IQR: 6–14) (*p* < 0.001) (Figure 5). More specifically, there was a reduction in motor signs according to question 1 (*p* = 0.001) and of motor complications according to question 3 (*p* < 0.001).

#### Daytime PKG score

Figure 4A presents the daytime PKG scores. Percent time tremor (PTT) showed a significant improvement with a median score of 2.1 (IQR: 0.9–9.2) before treatment and 1.7 (IQR: 0.7–4.8) with treatment (*p* < 0.001). Ten patients fell within the PTT target range (≤1%) before treatment, and the number increased to 13 with treatment. Median dyskinesia score (mDKS) and percent time in dyskinesia (PTD) increased after rotigotine treatment; mDKS from 0.95 (IQR: 0.5–2) to 1.6 (0.7–2.7) (*p* = 0.001), and PTD from 1.7 (IQR: 0–6.7) to 3.7 (IQR: 0.6–12.7) (*p* = 0.016). In one patient mDKS increased to >9 during rotigotine treatment, indicating uncontrolled dyskinesia that were not present before (34). There was a trend toward improvement in median bradykinesia score (mBKS) with a median of 27.9 (IQR: 24.6–31.6) before rotigotine and 26.5 (IQR: 23–30.8) with rotigotine (*p* = 0.106). Before rotigotine treatment 23 patients had mBKS scores >25 indicating uncontrolled bradykinesia, and in 4 of them (17%) mBKS ≤25 was achieved during rotigotine treatment. No patients deteriorated in BKS during treatment.

### Correlations between PKG scores and questionnaires

**Figure 6:**
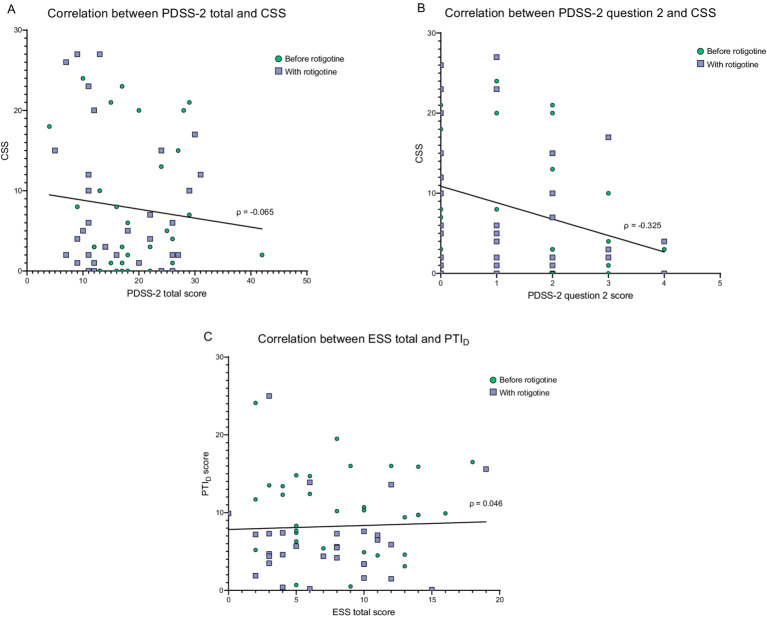
Correlation between PKG score and questionnaires. Green dots = before rotigotine treatment. Purple squares = with rotigotine treatment. The lines represent simple linear regressions. **(A)** Correlation between PDSS-2 total score and combined sleep score (CSS). PDSS-2 stands for Parkinson’s disease sleep scale 2, and CSS is measured with PKG. The Spearman correlation coefficient was −0.065 (*p* = 0.612). Simple linear regression gives the equation Y = −0.1115*X + 9.934 (*p* = 0.415). **(B)** Correlation between PDSS-2 question 2 (difficulty falling asleep) and combined sleep score (CSS). The Spearman correlation coefficient was −0.325 (*p* = 0.009). Simple linear regression gives the equation Y = −2.051*X + 10.88 (*p* = 0.0138). No significant correlations were identified between the CSS and any other PDSS-2 items. **(C)** Correlation between ESS total score and PTI_D_. ESS refers to the Epworth Sleepiness Scale, while PTI_D_ represents the percentage of time immobile during the daytime measured with PKG, which estimates daytime sleepiness. The Spearman correlation coefficient was 0.046 (*p* = 0.718). Simple linear regression gives the equation Y = 0.05145*X + 7.833 (*p* = 0.749).

No correlation was observed between total PDSS-2 and CSS (ρ = −0.065, *p* = 0.612) (Figure 6A). However, there was a negative weak correlation (ρ = −0.325, *p* = 0.009) between CSS and PDSS-2 question 2 (difficulty falling asleep; Figure 6B). No significant correlations were identified between the CSS and any other PDSS-2 items. There was no significant correlation between ESS and PTI_D_ (ρ = 0.046, *p* = 0.718) (Figure 6C). The PTI_D_ score was not significantly higher in patients with ESS score ≥10 compared with patients with an ESS score <10 (Mann Whitney Test; *p* = 0.967) and patients with PTI_D_ > 10 did not exhibit significantly higher ESS scores (Mann Whitney Test; *p* = 0.873).

## Discussion

Sleep problems, as assessed by PDSS-2, showed a trend toward improvement with rotigotine treatment, but the finding was statistically significant only in patients with more severe baseline sleep disturbances (PDSS-2 ≥ 18) and among those who were DA-naïve. Clinician assessments using the CGI-S scale indicated sleep improvement, while PKG nighttime scores remained unchanged. PTI_D_ improved, suggesting rotigotine to reduce daytime sleepiness. Furthermore, motor symptoms and quality of life improved significantly with rotigotine treatment, as indicated by CISI-PD, PTT, PDQ-8, and EQ-5D-5L scores. No significant correlations were observed between PTI_D_ and ESS or between CSS and PDSS-2.

While previous studies have reported positive effects of rotigotine on sleep (5, 22, 23, 35), our study found no significant improvements in sleep for the entire study group, as measured by PDSS-2 and PKG nighttime scores. This may be due to the use of the CGI-S scale for patient inclusion, which is less comprehensive than PDSS-2, along with a low severity threshold for inclusion. The CGI-S was selected as a quick screening tool to include patients with both severe and mild sleep disturbances. Previous studies investigating rotigotine’s effect on sleep used similar inclusion methods, facilitating comparison (3, 4). While PD-specific scales like PDSS-2 identify PD-related sleep issues more accurately, setting a PDSS-2 cutoff could exclude patients with significant sleep disturbances. For instance, a patient with severe insomnia but no other issues could receive a relatively low PDSS-2 score. The CGI-S scale indicated significant sleep improvement, unlike PDSS-2 or PKG scores. The CGI-S scale relies on patient reports, and patients may have exaggerated sleep improvements during clinician discussions but not on questionnaires. Also, CGI values change differently from PDSS-2, which weighs all changes equally and linearly, potentially not reflecting real-life experiences.

In this study, the mean treatment duration was 29 days, with an optimal rotigotine dose maintained for an average of 16 days (range: 13–32), with the second registration the last week of maintenance dose. While one other study used a 2 week maintenance period (23), most studies assessed sleep after 4 weeks at the optimal dose (3, 5). A longer treatment duration may be needed to fully evaluate rotigotine’s effects. The mean rotigotine dose was 5 mg (range: 4–8 mg), while previous studies have reported doses ranging from 2 mg to 18 mg (3, 4, 8), with many averaging around 8–9 mg (4, 5, 23). Higher rotigotine doses might produce different outcomes. Few studies have assessed rotigotine’s dose-dependent effects on sleep. Anthony et al. (36) suggested 8 mg as the minimum dose to reduce “off” time but found no improvements in ESS or PDSS across 2–8 mg. While some studies reported sleep benefits at doses as low as 2 mg (13, 14), most showing improvements used higher doses (4, 5, 13, 23). The adaptive dose regimen in the current study was not specifically designed to address sleep problems, but the final dosing provides some indication about a dose threshold for positive effects in general. Further research is needed to clarify the dose–response relationship for sleep outcomes in PD.

The median baseline PDSS-2 score was 17, with 47% of patients scoring ≥18 before treatment. A cutoff of 18 points on the PDSS-2 is suggested as a marker for clinically relevant PD-specific sleep disturbances (33). Improvements in the PDSS-2 total score and the subtotals for “disturbed sleep” and “PD symptoms at night” were observed only in patients with a baseline PDSS-2 score of ≥18. These findings suggest that rotigotine’s sleep-enhancing effects are more pronounced in patients with severe sleep issues, consistent with Vallderiola et al. (37), who found that higher baseline PDSS-2 scores correlated with greater improvement of PDSS-2 score after 3 months of night-time rotigotine treatment. Identifying the patient groups whose sleep improves with rotigotine is crucial for physicians when determining who should be offered the treatment.

While all participants were evaluated without DA treatment at baseline, half had previously used oral DA. Subgroup analysis showed significant improvements in PDSS-2 total score for DA-naïve patients, while those with earlier oral DA showed no significant improvement. This contradicts Pagonabarraga et al. (4), who found no differences in PDSS-2 between DA- naïve patients and those switching from another DA to rotigotine. Possible explanations for this discrepancy include variations in sample sizes, differing durations of previous DA treatment, and individual patient characteristics. Further research is needed to assess rotigotine’s effect on sleep in relation to prior DA use and concurrent therapies.

No significant correlation was found between PDSS-2 and CSS, though a negative correlation was observed between CSS and PDSS-2 question 2 (difficulties falling asleep), indicating that CSS may be primarily influenced by sleep onset insomnia. Previous research has shown that CSS correlates with PDSS-2, especially in subscores related to sleep quality and quantity (18). PDSS-2 scores reflect contributing factors to sleep disturbances rather than sleep extent or quality, and the non-linear nature of PDSS-2 complicates the interpretation of correlation patterns (18). Thus, a strong linear correlation between CSS and PDSS-2 scores is unlikely. However, to support the reliance on PKG nighttime scores, a correlation between worsening PDSS-2 scores and CSS deterioration is expected, and such correlation was not seen in this study. The lack of correlation may be attributed to the subjective nature of the PDSS-2 or the need for a larger sample size. PSG is the gold standard for sleep assessment (15), and comparisons between PKG and PSG have suggested PKG to be a valuable tool for assessing sleep (18). Further studies are needed to determine the reliability of PKG nighttime scores for assessing sleep in PD patients, ideally incorporating both PSG and questionnaire assessments.

The reduction in the PKG PTI_D_ score, along with the normalization of PTI_D_ scores in 12 patients, suggests that rotigotine improves daytime immobility and potentially reduces daytime sleep episodes. Unlike other DAs, rotigotine has not been shown to worsen EDS or somnolence (11–13). Calandra-Buonara et al. (5) demonstrated through actigraphy that rotigotine decreased number and duration of daytime sleep episodes, while Steiger (38) observed a 70% reduction in daytime sleepiness with continuous dopaminergic treatment. Additionally, an open label study reported improvements in ESS score after 1 and 3 months of rotigotine treatment (14). These findings support the notion that rotigotine, with its continuous dopaminergic treatment (39), may have a positive effect on daytime sleepiness. Although PTI_D_ improved with rotigotine treatment, no significant change was observed in ESS score. This aligns with findings by Calandra-Buonara et al. (5), who found improved daytime sleepiness via actigraphy with rotigotine but no change in ESS scores. The subjective nature of the ESS, along with patients’ potential difficulty recalling symptoms over the past week and a tendency to under-report sleepiness due to unawareness of daytime naps, may affect its accuracy (40).

No significant correlation was found between PTI_D_ and ESS. Although an ESS score ≥10 indicates EDS (26), and Kotschet et al. (20) reported a significant association between ESS scores ≥10 and elevated PTI_D_ scores in PD patients, we observed no differences in PTI_D_ scores between patients with ESS scores above or below 10. Similarly, Höglund et al. (21) found no correlation between PKG score and self-evaluated daytime sleepiness. The lack of correlation between ESS and PTI_D_ may stem from the subjective nature of questionnaires, where patient ratings can vary despite similar symptoms. Also, one study showed that over a third of both PD patients and healthy individuals underreport brief naps with slow-wave sleep (40). Additionally, PTI_D_ measures immobility, and the CISI-PD scale indicates that rotigotine improved motor symptoms, likely leading to reduced immobility. Therefore, part of the discrepancy between ESS and PTI_D_ scores could, to some extent, be explained by PTI_D_ reflecting bradykinesia and immobility caused by motor symptoms, rather than daytime sleepiness. Larger studies comparing PKG data with questionnaires and PSG are needed to better evaluate PKG’s effectiveness in assessing sleep and daytime sleepiness in PD patients.

PTI_D_ improved with rotigotine treatment despite no improvement of nighttime PKG scores. Supporting this, Klingelhoefer et al. (19) found that nighttime PKG variables do not differentiate between patients with EDS and without EDS, and Liguori et al. (12) found no correlation between nocturnal sleep and daytime sleepiness. Conversely, McGregor et al. (18) showed that more severe daytime sleepiness was associated with poorer nighttime PKG scores. It is possible that daytime sleepiness can occur independently of nighttime sleep disturbances, though sleep disruptions likely contribute to it. Further research with multiple objective tools is needed to clarify the relationship between sleep disturbances and daytime sleepiness in PD.

Significant improvements in CISI-PD and PTT were observed with rotigotine treatment. Also, four patients who previously had uncontrolled bradykinesia according to the PKG achieved control within PKG target ranges (18, 34). These findings suggests that rotigotine reduces motor symptoms in PD patients, consistent with prior studies (35, 41). While PTD and mDKS increased significantly with rotigotine, they remained at low levels of clinically non-significant dyskinesia. Additionally, rotigotine significantly improved quality of life as measured by PDQ-8, and EQ-5D-5, aligning with previous research (3, 37).

This study has some limitations. A larger sample size could have improved the reliability of the results, and a longer treatment period or higher rotigotine doses might have led to different outcomes. Subjective questionnaires can be interpreted differently by patients and clinicians, potentially affecting the result. However, the same clinician rated the patient both before and with treatment, which is a strength. Moreover, specific tools for evaluating other sleep disturbances, such as restless legs syndrome and rapid eye movement sleep behavior disorders, were lacking. Also, while CISI-PD was used to assess motor symptoms, a more detailed scale like the Movement Disorder Society-Unified Parkinson’s disease rating scale might have offered richer clinical information. The lack of a placebo or control group introduces bias, as expectations may have influenced symptom assessments, which a double-blind design could have mitigated. Moreover, the observational study design led to a heterogeneous study group with varying PD duration, LED doses, and ages may have further impacted results, and a more homogeneous cohort might yield different findings. Additionally, using PSG to validate PKG and exclude patients with sleep apnea would have been preferable. However, it is a strength that the study investigated sleep with both subjective and objective tools. Furthermore, half of the patients had previously used a DA, which was tapered off and discontinued at least 28 days prior to study inclusion, to ensure comparability across baseline visits. This washout period may have altered baseline conditions, potentially influencing the findings.

## Conclusion

In conclusion, while rotigotine did not significantly improve sleep in the entire group based on PDSS-2 or PKG nighttime scores, sleep improved in those with clinically relevant sleep disturbances (PDSS-2 ≥ 18) and in DA-naïve patients. Moreover, PKG measures showed reduced daytime immobility with rotigotine, suggesting a potential reduction in daytime sleepiness. Confirming this in future studies could establish rotigotine as a promising option for PD patients with daytime sleepiness. Additionally, rotigotine improved motor symptoms and quality of life. No correlations were seen between CSS and PDSS-2 or between ESS and PTI_D._ Larger studies are needed to further explore correlations between PKG scores, PSG, and established rating scales.

## Data Availability

The raw data supporting the conclusions of this article will be made available by the authors, without undue reservation.
