# Supplementary material for: Effects of rotigotine on sleep in Parkinson’s disease patients: a Parkinson’s KinetiGraph study
Source: Front Neurol. 2025 May 27;16:1591537. doi: 10.3389/fneur.2025.1591537 (PMC12150402; doi:10.3389/fneur.2025.1591537)
Supplement: Supplementary file 1 [file Table_1.DOCX]

**Glossary of PKG terms**

*Epoch:* Each 2-min period of recording is called an epoch (1).

*Bradykinesia score (BKS):* BKS is calculated for each epoch during PKG wear, with higher scores indicating greater severity of motor problems and bradykinesia (2). The daytime median BKS represents the median of epochs BKS scores <80 between 09.00 and 18.00. A BKS >80 is associated with daytime sleep, therefore BKS = 80 was set as the boundary between wakefulness and sleep (3). Controlled bradykinesia is defined as mBKS ≤25 (3, 4).

*Percent time in bradykinesia (PTB):* PTB represents the proportion of time a patient experiences uncontrolled bradykinesia (mBKS_D_ >25), indicating an “Off” state. (1)

*Percent time in tremor (PTT):* PTT represents the percentage of epochs between 9:00 and 18:00 that exhibit tremor. If PTT is >1% tremor is likely to be present. (5)

*Dyskinesia score (DKS):* The DKS is calculated for each epoch during PKG wear. The mDKS represents the median value of DKS between 09.00 and 18.00 (3). A DKS ≤9 is defined as controlled (4).

*Percent time in dyskinesia (PTD):* Is calculated between 09.00 and 18.00 and is estimated as the percent time of epochs whose dyskinesia score ≥ 10 and in which neither walking nor tremor is detected (1).

*Percent time immobile (PTI):* PTI_D_ is a measure of daytime immobility that has shown concordance with the detection of sleep by polysomnography (PSG) (6).High PTI values have been associated with higher ESS scores in PD patients (6). PTI_D_ is defined as episodes of immobility (bradykinesia score (BKS) >80) between 09.00 and 18.00, lasting for at least 2 minutes (2, 3). The target score is ≤10% (5). PTI_N_ is the percent of BKS>80 between 23:00 and 06:00, providing an estimate of sleep efficiency. A study showed PTI_N_ in healthy controls to be 69% (IQR 14). A higher score indicates better sleep (3).

*Percent time sleep (PTS):* PTI may underestimate sleep due to PKG misidentifying periodic limb movements and micro arousals as wakefulness. PTS is calculated with a smoothing function that reduces the impact of these factors, providing a more accurate measure of sleep efficiency (3). Nighttime PTS (PTS_N_) is calculated between 23:00 and 06:00. Controls exhibited a median PTS_N_ of 64% (IQR = 18) (3).

*Night percent Time Active (PTA):* PTA is calculated as the percentage of epochs between 23:00 and 06:00 with a BKS score <80, which is indicative of being awake. In controls, the median has been shown to be 31% (IQR = 14) (3).

*Sleep Fragmentation (Sleep Frag):* The median duration (in minutes) of fragmented sleep between 23:00 and 06:00. A sleep fragment is defined as a period with BKS >80, and the length of the fragment is determined by the number of BKS in that sequence. Knowledge of sleep fragmentation provides insights into sleep quality. In control subjects, the median has been reported as 38 minutes (IQR = 33) (3)

*Sleep Quality (SQ):* A BKS >110 correlates with deeper sleep in 80% of instances. Thus, the percentage of the night period (23:00 - 06:00) with BKS>110 is used as an indicator of sleep quality. In controls, the median is estimated as 77% (IQR = 15). Higher scores denote better sleep quality. (3)

*Combined sleep score (CSS):* PTI_N_, PTS and SQ variables are normalised according to their percentile ranking (0–10th percentile, '0'; 10–20th percentile, '1'; etc. to 90–100th percentile, '9') and transformed into scores ranging from 0 to 9, with higher scores indicating better sleep. The CSS score is calculated by combining PTI_N_ score, PTS_N_ (percent time sleep during night-time) score and SQ (sleep quality) score. CSS is the sum of these scores with a maximum of 27. A higher CSS value indicates better sleep, and CSS has been shown to correlate with PDSS 2. (3)

**References**

1. Khodakarami H, Shokouhi N, Horne M. A method for measuring time spent in bradykinesia and dyskinesia in people with Parkinson's disease using an ambulatory monitor. J Neuroeng Rehabil. 2021;18(1):116-.

2. Farzanehfar P, Woodrow H, Braybrook M, McGregor S, Evans A, Nicklason F, et al. Objective measurement in routine care of people with Parkinson’s disease improves outcomes. npj Parkinson's Disease. 2018;4(1):10.

3. McGregor S, Churchward P, Soja K, O’Driscoll D, Braybrook M, Khodakarami H, et al. The use of accelerometry as a tool to measure disturbed nocturnal sleep in Parkinson’s disease. npj Parkinson's Disease. 2018;4(1):1.

4. Pahwa R, Isaacson S, Torres-Russotto D, Nahab F, Lynch P, Kotschet K. Role of the Personal KinetiGraph in the routine clinical assessment of Parkinson’s disease: Recommendations from an Expert Panel. Expert Review of Neurotherapeutics. 2018;18.

5. Pahwa R, Bergquist F, Horne M, Minshall ME. Objective measurement in Parkinson’s disease: a descriptive analysis of Parkinson’s symptom scores from a large population of patients across the world using the Personal KinetiGraph®. Journal of Clinical Movement Disorders. 2020;7(1):5.

6. Kotschet K, Johnson W, McGregor S, Kettlewell J, Kyoong A, O'Driscoll DM, et al. Daytime sleep in Parkinson's disease measured by episodes of immobility. Parkinsonism Relat Disord. 2014;20(6):578-83.
